# Factors associated with the contraindicated use of oral contraceptives in Brazil

**DOI:** 10.1590/S1518-8787.2017051006113

**Published:** 2016-12-19

**Authors:** Daniele Aparecida Silva Corrêa, Mariana Santos Felisbino-Mendes, Mayara Santos Mendes, Deborah Carvalho Malta, Gustavo Velasquez-Melendez

**Affiliations:** I Programa de Pós-Graduação em Enfermagem. Escola de Enfermagem. Universidade Federal de Minas Gerais. Belo Horizonte, MG, Brasil; II Departamento de Enfermagem Materno-Infantil e Saúde Pública. Escola de Enfermagem. Universidade Federal de Minas Gerais. Belo Horizonte, MG, Brasil

**Keywords:** Contraceptives, Oral, contraindications, Prescription Drug Misuse, Risk Factors, Women’s Health, Sexual and Reproductive Health

## Abstract

**OBJECTIVE:**

To estimate the prevalence of the contraindicated use of oral contraceptives and the associated factors in Brazilian women.

**METHODS:**

20,454 women who answered the VIGITEL survey in 2008 also participated in this study, of which 3,985 reported using oral contraceptives. We defined the following conditions for the contraindicated use of contraceptives: hypertension; cardiovascular diseases such as heart attack, stroke/cerebrovascular accident; diabetes mellitus; being smoker and 35 years old or older. We estimated the prevalence and 95% confidence intervals of contraindicated use in users of oral contraceptives and the factors associated with contraindication by prevalence ratio and 95% confidence intervals.

**RESULTS:**

In the total population, 21% (95%CI 19.7–21.9) of women showed some contraindication to the use of oral contraceptives, of which 11.7% (95%CI 10.6–13.7) belonged to the group of users of oral contraceptives. The most frequent contraindication in users of oral contraceptives was hypertension (9.1%). The largest proportion of women with at least one contraindication was aged between 45 and 49 years (45.8%) and with education level between zero and eight years (23.8%). The prevalence of contraindication to oral contraceptives was higher in women less educated (zero to eight years of study) (PR = 2.46; 95%CI 1.57–3.86; p < 0.05) and with age between 35-44 years (PR = 4.00; 95%CI 2.34–6.83) and 45-49 years (PR = 5.59; 95%CI 2.90–10.75).

**CONCLUSIONS:**

Age greater than or equal to 35 and low education level were demographic and iniquity factors, respectively, in the contraindicated use of oral contraceptives.

## RESUMO

**OBJETIVO:**

Estimar a prevalência de contraindicação ao uso de anticoncepcionais orais e os fatores associados em mulheres brasileiras.

**MÉTODOS:**

Participaram 20.454 mulheres que responderam ao inquérito Vigitel em 2008, das quais 3.985 reportaram uso de contraceptivos orais. Definiu-se como uso contraindicado de anticoncepcionais quando presente pelo menos uma condição: hipertensão; doenças cardiovasculares como infarto, derrame/acidente vascular encefálico; diabetes mellitus; ser tabagista e ter idade igual ou maior de 35 anos. Foram estimadas as prevalências e intervalos de 95% de confiança de uso contraindicado em usuárias de anticoncepcionais orais e fatores associados à contraindicação por meio de razões de prevalência e intervalos de 95% de confiança.

**RESULTADOS:**

Na população total, 21,0% (IC95% 19,7–21,9) das mulheres apresentaram alguma contraindicação ao uso de anticoncepcionais orais, das quais 11,7% (IC95% 10,6–13,7) pertenciam ao grupo de usuárias de anticoncepcionais orais. A contraindicação mais freqüente entre as usuárias de anticoncepcionais orais foi hipertensão (9,1%). A maior proporção de mulheres com pelo menos uma contraindicação tinha entre 45 a 49 anos (45,8%) e escolaridade entre zero e oito (23,8%). A prevalência de contraindicação de anticoncepcionais orais foi maior nas mulheres menos escolarizadas (zero a oito anos de estudos) (RP = 2,46; IC95% 1,57–3,86; p < 0,05) e idade entre 35-44 anos (RP = 4,00; IC95% 2,34–6,83) e 45-49 anos (RP = 5,59; IC95% 2,90–10,75).

**CONCLUSÕES:**

Idade maior ou igual a 35 e escolaridade baixa foram fatores demográficos e de iniquidade, respectivamente, no uso contraindicado de contraceptivos orais.

## INTRODUCTION

About 60% of women in reproductive age use some contraceptive method^1^. This prevalence reaches about 70% in Brazil, and oral contraceptive pills (OCP) and female sterilization are the most common methods (23.0%)^a^.

The OCP, when used correctly and continuously, give the woman effective and safe control of her fertility^11^ ^,^ ^21^. On the other hand, an online survey conducted in eight countries showed discontinuation rate of use of the method of 81%, mostly due to the adverse effects of the medicine (57%)^8^. The same study noted forgetfulness (65%) and intake in wrong time (67%) as frequent problems between users of the pills in the Country.

Even before the many benefits that this method can offer, such as regularization of the menstrual cycle and prevention of some types of cancer^12^, the use of OCP along with some conditions such as high blood pressure can increase the risk of cerebrovascular accident (CVA), acute myocardial infarction (AMI), and other adverse outcomes in women^5^. Besides hypertension, the following conditions contraindicate the use of OCP: diabetes mellitus with vascular disease, smoking in women with 35 years old or older, cardiovascular diseases, thromboembolism, migraine with aura, among others. In response to this situation, the Brazilian Ministry of Health and other international agencies adopted recommendations for their use^b^ ^,^ ^c^. The eligibility criteria for the use of OCP can be determined by a detailed evaluation, with clinical and family history of the woman and blood pressure measurement. In some countries, access to the method is subject to previous evaluation. In the United States, in addition to the contraindications being infrequent in women in reproductive age, the prevalence of use along with contraindication is approximately 5%^5^ ^,^ ^18^ ^,^ ^21^.

One way to acquire or start the use of OCP in Brazil is by consultation with health professional in public or private health services. Another possibility is the acquisition of the drug in drugstores without mandatory prescription.

Due to the widespread use of OCP without prescription, the ignorance of the contraindicated use is very likely. This can lead to adverse health effects. A recent study assessed the knowledge of women on the effects of OCP on their health in five Brazilian cities and showed that users know little about the contraceptive method they are using^11^.

There are no Brazilian population surveys to assess the extent of this problem. A few studies show concern in assessing the presence of contraindications to the use of the pill among women^5^ ^,^ ^18^ ^,^ ^21^ ^,^ ^22^. This is relatively expected in Brazil, since most women is not under the control of health services, acquiring the pill directly from drugstores.

Knowing the trends of use of contraceptive methods and their associated factors, as well as the characteristics of women using the method, can contribute to the planning and adaptation of public policies and to better access and use by the population.

This study aimed to estimate the prevalence of contraindicated use of OCP in Brazilian women. Our hypothesis is that there are differences in prevalence of contraindicated use of OCP according to sociodemographic variables in Brazilian women.

## METHODS

This study used the rotating module of questions related to the use of contraceptive methods answered by women who participated in the system of *Vigilância de Fatores de Risco e Proteção para Doenças Crônicas por Inquérito Telefônico* (VIGITEL) in 2008. It is composed of probabilistic samples of the adult population living in households with at least one fixed telephone line in the current year in the capitals of the 26 Brazilian States and in the Brazilian Federal District^13^.

The questionnaire included questions about feeding, physical activity, smoking, alcohol consumption, self-reported morbidity, weight, and height, health situation, and sociodemographic variables. In 2008, rotating modules were incorporated in the questionnaire: possession of health insurance, access to mental health services, and use of contraceptive methods. The following questions were included: “Currently, do you use any method to prevent pregnancy?” (yes/no); “Which method do you use more frequently?” (tubal ligation, condom, injection, pill, hormonal implant, IUD, diaphragm, vasectomy – for men only –, others).

The VIGITEL interviewed 54,353 individuals in 2008, of which 32,918 were women. Of these, 21,074 had reproductive age (15 to 49 years) and constituted the population of this study (Figure).

**Figure:**
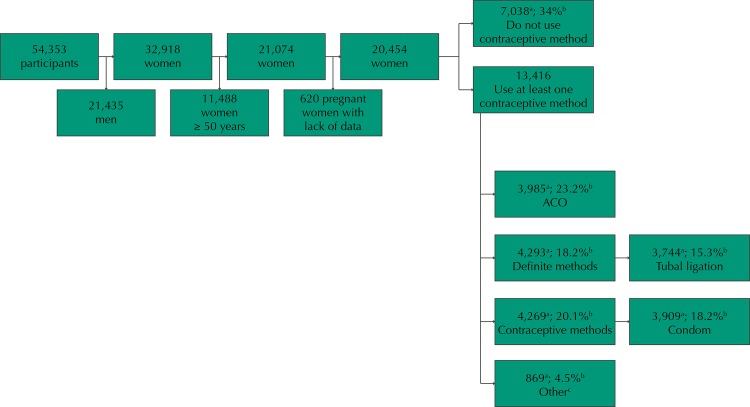
Flowchart of the population available for study. VIGITEL, Brazil, 2008.

OCP: oral contraceptive pills

^a^ sample n.

^b^ population estimate.

^c^ intrauterine device and diaphragm.

Exclusion criteria were: women who reported being pregnant at the time of interview (n = 457) or who did not know (n = 112); and women who have not responded any alternative of the covariates of interest, such as having access to health insurance (n = 28) and skin color (n = 21), totaling 20,454 women. Of these, 34% reported not carrying out family planning or using any contraceptive method. Compared to those who reported using some method, 23.2% reported use of oral contraceptives (Figure).

The dependent variables were constructed using the definition of contraindication to the use of oral contraceptives published in 2010 by the World Health Organization (WHO) and adopted by the Brazilian Ministry of Health (MS)^c^, as well as the more recent recommendation of the Centers for Disease Control in the United States^b^. We considered the following conditions for the contraindicated use of contraceptives: hypertension (SH) (yes/no); cardiovascular diseases (CVD) such as heart attack and stroke/CVA (cerebrovascular accident) (yes/no); diabetes mellitus (yes/no); being smoker and over 35 years old at the same time (yes/no). The occurrence of at least one of these conditions was considered a contraindication to the use of OCP.

The independent variables used were: age (18 to 24, 25 to 34, 35 to 44, 45 to 49 years), education level (zero to eight, nine to 11, 12 or more years of study), skin color (white, black, brown, and others), marital status (single, married or in stable union, widow, divorced), health plan or insurance (yes/no), housing region (North, Northeast, South, Southeast, and Midwest), all self-reported.

We conducted a non-conditional analysis based on the definition of subpopulation of interest^19^. Missing data and subgroups that met exclusion criteria were included in the category 0 of the variable indicating subpopulation, and the studied population was included in category 1. This method is more suitable to estimate the variance for representative samples of large populations. We estimated the prevalence and 95% confidence intervals (95%CI) of the contraindications for all population and according to the use of contraceptives. For women who reported using OCP, we estimated the proportions and non adjusted prevalence ratios (PR) and 95%CI of the use of OCP along with at least one contraindication, according to sociodemographic characteristics, using Poisson regression. Statistical differences between the proportions were evaluated by Pearson’s Chi-squared test. We estimated the adjusted PR and 95%CI. We used Wald’s Chi-squared test to assess the statistical significance of the contribution of each variable in the multivariate model. A significance level of p < 0.05 was used to reject the null hypothesis in all tests.

We used the statistical program Stata, version 12.0, and the commands of the survey module, considering the weights and representativeness of the sample.

The VIGITEL was approved by the National Research Ethics Committee for Human Beings of the Ministry of Health under Opinion 749/2006, according to the principles of the Declaration of Helsinki. As the data were obtained by telephone interview, the informed consent form was replaced by verbal consent, obtained during the telephone contacts with respondents. The researchers did not have access to the identity of participants.

## RESULTS

Of 20,454 women, about 21.0% showed at least one potential contraindication to use OCP, with 11.7% of them in the group of users and 23.5% in the group of non-users (Table 1).

**Table 1:** Prevalence of contraindications to oral contraceptive use among Brazilian women. VIGITEL, Brazil, 2008.

Contraindications	Total women (n = 20.454)			Use of OCP					

				Yes (n = 3.985)			No (n = 16.469)		
		
	n	%	95%CI	n	%	95%CI	n	%	95%CI
At least one contraindication	4,040	20.8	19.7–21.9	480	11.7	10.6–13.7	3,560	23.5	22.3–24.8
Number of contraindications									
1	3,454	17.4	16.4–18.4	426	10.5	8.9–12.4	3,028	19.5	18.4–20.7
2	531	3.1	2.6–3.7	50	1.2	0.8–1.9	481	3.6	3.0–4.3
3 or +	55	0.3	0.1–0.5	4	0.03	0.01–0.1	51	0.4	0.2–0.6
Types of contraindications									
SH	2,874	15.1	14.1–16.1	354	9.1	7.6–10.8	2,520	16.9	15.7–18.1
Smokers and ≥ 35 years	1,156	6.1	5.5–6.7	112	2.6	1.8–3.7	1,044	7.1	6.4–8.0
DM	451	2.2	1.8–2.6	51	0.9	0.6–1.4	400	2.6	2.2–3.1
CVD	201	1.1	0.8–1.5	21	0.5	0.3–0.9	180	1.3	0.9–1.8

OCP: oral contraceptive pills; SH: systemic hypertension; DM: diabetes mellitus; CVD: cardiovascular disease

Among OCP users, 10.5% presented one contraindication and 1.2%, two contraindication conditions (Table 1). The most frequent contraindication was systemic hypertension (SH) (15.1%). The habit of smoking in women aged 35 years or older was the second most frequent contraindication (2.6%) (Table 1).

There was higher prevalence of contraindicated use in women who have low education level (zero to eight years of study), when compared with those with more education (PR = 3.76; 95%CI 2.44–5.80) (Table 2). This prevalence was also high in women aged 45-49 years when compared with younger ones (PR = 7.30; 95%CI 4.2–12.8). The contraindicated use of OCP was more frequent in separated/divorced women (27.3%), followed by widow women (15%). A higher proportion of women presented at least one contraindication among those who had health plan or insurance (14.8%) (p < 0.05). The contraindication was more prevalent in women classified as other (yellow, red/indigenous, do not know, and would not inform) (21%), followed by brown (13.6%) and black women (11.2%). The North region concentrated most women with at least one contraindication (14%), followed by South (11.9%) and Southeast (11.8%).

**Table 2:** Prevalence and 95% confidence intervals of contraindicated use of oral contraceptive among users of the method, according to sociodemographic characteristics. VIGITEL, Brazil, 2008.

Sociodemographic characteristics	Women using OCP						

	n	%	95%CI	p^a^	PR	95%CI	p^b^
Education level (years)				< 0.0001			
12 and more	1,840	6.3	4.4–9.0		1.0	-	-
9 to 11	1,670	10.4	8.2–13.0		1.6	1.1–2.5	0.022
0 to 8	475	23.8	18.5–30.0		3.8	2.4–5.8	< 0.000
Age (years)				< 0.0001			
18 to 24	1,172	6.2	3.4–9.6		1.0	-	-
25 to 34	1,858	7.5	5.7–9.7		1.2	0.7–2.0	0.493
35 to 44	823	30.7	25.0–37.0		4.9	3.0–8.0	< 0.000
45 to 49	132	45.8	31.3–61.1		7.3	4.2–12.8	< 0.000
Marital status				0.0001			
Single	2,021	8.8	6.8–11.3		1.0	-	-
Married	1,802	14.8	12.1–18.1		1.7	1.2–2.3	0.001
Widow	26	15.0	5.3–35.8		1.7	0.6–4.7	0.303
Separated/Divorced	136	27.3	15.7–43.1		3.1	1.8–5.5	< 0.000
Has health plan or insurance				0.003			
Yes	2,308	14.8	12.2–17.7		1.0	-	-
No	1,677	9.2	7.1–11.9		0.6	0.4–0.8	0.004
Skin color				0.176			
White	1,832	9.9	7.7–12.5		1.0	-	-
Black	190	11.2	6.6–18.5		1.1	0.6–2.0	0.660
Brown	1,942	13.6	11.0–16.8		1.4	1.0–1.9	0.530
Other	21	21.0	5.0–57.2		2.1	0.6–7.8	0.255
Region				0.844			
North	928	14.0	10.3–18.9		1.0	-	-
Northeast	979	11.5	8.8–15.0		0.8	0.5–1.3	0.334
Midwest	722	10.7	8.3–13.7		0.8	0.5–1.1	0.179
Southeast	734	11.8	9.0–15.3		0.8	0.6–1.2	0.394
South	632	11.9	9.2–15.2		0.8	0.6–1.2	0.409

OCP: oral contraceptive pills; PR: prevalence ratio

^a^ Pearson’s Chi-squared.

^b^ Wald’s Chi-squared.

The factors that remained associated with contraindications in women using OCP were low education level and age ≥ 35 years in the adjusted analysis (Table 3).

**Table 3:** Prevalence ratios and 95% confidence intervals for the presence of at least one contraindication among users of oral contraceptive, according to sociodemographic characteristics. VIGITEL, Brazil, 2008.

Sociodemographic characteristics	Contraindication		

	Adjusted PR	95%CI	p*
Education level (years)			
12 and more	1.0	-	-
9 to 11	1.7	1.1-2.5	0.014
0 to 8	2.5	1.6-3.9	< 0.000
Age (years)			
18 to 24	1.0	-	-
25 to 34	1.1	0.6–1.9	0.727
35 to 44	4.0	2.3–6.8	< 0.000
45 to 49	5.6	2.9–10.7	< 0.000
Marital status			
Single	1.0	-	-
Married	1.0	0.7-1.4	0.939
Widow	0.6	0.2-1.8	0.354
Separated/Divorced	1.5	0.8-2.8	0.160
Has health plan or insurance			
Yes	1.0	-	-
No	0.9	0.7–1.2	0.522
Skin color			
White	1.0	–	-
Black	0.9	0.5–1.7	0.754
Brown	1.1	0.8–1.6	0.450
Other	2.4	0.7–8.6	0.162
Region			
North	1.0	-	-
Northeast	0.8	0.5–1.2	0.522
Midwest	0.8	0.6–1.2	0.316
Southeast	0.8	0.6–1.3	0.444
South	0.8	0.6–1.2	0.353

PR: Prevalence Ratio

* Wald’s Chi-squared.

## DISCUSSION

About 20% of Brazilian women showed some condition that contraindicates the use of OCP. This proportion was 11.7% in women using OCP. The main condition for contraindicated use was SH, followed by age over 35 years along with smoking.

Most studies that measured the magnitude of use of OCP in the presence of contraindication were carried out with North American populations^5^ ^,^ ^18^ ^,^ ^21^ ^,^ ^22^. Data from the National Health and Nutrition Examination Survey (NHANES) showed contraindication prevalence of 6% among users of OCP in the United States, half of what we found, and 19% among non-users of the method^18^. Another study showed higher prevalence of contraindication among American women who buy OCP on drugstores (13%) when compared to those served in clinics (9%)^5^. However, an internet survey showed that 23.7% of North American users of OCP had some contraindication condition^22^. On the other hand, the North American Project CHOICE showed that 3% of women used OCP with some contraindication, showing great variability in the estimation of this parameter, which could be explained by the different samples and contexts of the women accessed in each study.

There are no similar population studies using a representative sample population in Brazil. A local study, conducted in Pelotas (RS), between 1992 and 1999, showed that, among the 279 users of OCP, 22.2% presented some contraindication for their consumption. The most prevalent condition was smoking in women over 35 years, followed by SH^3^.

In this study, the prevalence of contraindications to use OCP was similar to that of the presented studies. Thus, even if the use of OCP is safe as a measure of fertility control, their use along with a contraindication is an indicator of problems in the quality of actions for family planning and integrality of health actions. The sharp growth in the use of contraceptive methods in Brazil in recent decades reached about 80% of women in reproductive age in 2006^a^. Among the methods, the use of OCP is expected to keep growing, since they are the second most widely used contraceptive method in Brazil, followed by tubal ligature, often associated with the epidemic of Caesarean sections in force since the 90s. One must expect a behavior similar to the world trend, in which the OCP method may be the first election. Thus, the promotion of proper use is essential, that is, that the method is used by women who meet the eligibility criteria. The use along with a contraindication may result in damages to health, putting it at risk of more serious events.

Self-reported SH was the most prevalent contraindication, confirming studies carried out with other populations^5^ ^,^ ^21^. Women whose blood pressure was not measured before starting the use of OCP showed increased risk of acute myocardial infarction (AMI). Therefore, their blood pressure must be measure before starting the use of the method^20^ ^,^ ^c^. The prolonged use of OCP may increase twice the chance of developing hypertension^15^. This indicates that hypertension can be aggravated with the use of OCP, tripling the odds ratio (OR = 2.67) of non-control of blood pressure (≥ 140/90 mmHg) when compared to non-users^10^. On the other hand, the suspension of OCP in hypertensive women reduced in 15 mmHg the systolic blood pressure and in 10 mmHg the diastolic blood pressure^10^. In this study, we observed higher proportion of contraindicated use among women over 35 years, known to be more vulnerable to the occurrence of hypertension and other diseases, more prevalent with age.

The second most prevalent contraindication was smoking and age ≥ 35 years together. The risk of AMI in women who smoke and have less than 35 years is 10 times greater than that of those who do not smoke^20^, and this risk in women older than 35 is higher regardless of smoking. Thus, the risk of AMI among these users also increases with the coexistence of cardiovascular risk factors, such as smoking, which potentiates this effect on women over 35^2^ ^,^ ^20^. The number of cigarettes also affects this evaluation, but this information is not available on VIGITEL, 2008.

The contraindicated use of OCP is also a cardiovascular risk factor, especially in women over 30 years old. This is troubling, since the main cause of morbidity and mortality in Brazil are the cardiovascular system diseases^d^. Facing this problem means preventing risk factors, including the contraindicated use of OCP^3^.

This study showed that women over 34 years, separated/divorced, with private health plan, and low education level presented higher prevalence of contraindication to the use of OCP. Explanations of these findings as a whole are relatively complex, since they involve factors of diverse and sometimes speculative nature, such as access to information and effective management of contraception, which involves issues such as the criterion of effectiveness, unimpeded sexuality, and female protagonism that the use of the pill represents to many women^e^. In addition, there is reproductive assistance concentrated in women who experience the pregnancy-puerperal cycle^7^, leaving women with extremes of age, as adolescents and older women, on the sidelines of the reproductive planning. Self-medication may still be a result of individual social and political processes, not necessarily of access to method and information^4^. It can also result from feminine socialization marked by learning with friends, from the acquisition of the medicine in drugstores, and from the dependence of this for contraception^e^. Finally, the pill was reported as an easier method to use and obtain in health services^e^. However, a regional study showed that neither the indication for use of OCP in health services nor getting the medicine on the service itself have improved its fitness when compared with the acquisition by the user herself^6^. We were not able to show this result here due to the lack of data about the source of recommendation of its use.

Education level was an iniquity factor to the use of OCP: there was higher prevalence of contraindication in women with low educational level. The education level is expected to improve the proper choice of OCP. Better educated women used contraceptive methods more often. Those with less education had more children than they wanted to, and the most prevalent method was tubal ligation^d^. As is the case in Brazil, in high-income countries, the difficulty of choice of appropriate method can be extended to women who did not finish higher education^22^. Women with more education may have greater understanding of information about the method they wish to use and the risks inherent in it.

The unequal access to contraception can also be related to increased access to the diversity of methods by women with more education. The findings point to the difficulty of SUS in achieving greater democratization of access to the wide variety of contraceptive methods, as well as to the inefficiency in the supply of reproductive planning assistance in primary health-care.

One of the main limitations of this study is the use of telephone interviews. The coverage of fixed telephone lines ranges from 34% to 82% in the capitals in which the samples were drawn. However, the system presents response rates above 60%. Because of its convenience and relatively low cost, the use of health surveillance systems by telephone survey is an efficient and practical methodology to the context of health surveillance. A comparative study between researches conducted by face-to-face interviews and telephone survey provides evidence in favor of the latter, provided that it is performed with well-structured questionnaires and trained interviewers^14^ ^,^ ^17^. This limitation can be circumvented with the inclusion of post-stratification weights of the samples. The use of post-stratification corrections obtained by the weights estimated using statistical strategies effectively reduces the risk of systematic errors related to fixed telephony coverage.

Another limitation of this study is the self-reported data, since subclinical events may be underestimated. This can be due to ignorance of the health conditions of the individual, as a result of many reasons, such as lack of access to health services, lack of consensus among doctors about the criteria used to report these conditions to the bearer, individual limitations regarding the capacity of understanding of individuals about their own health condition.

Some important contraindications to the use of OCP, such as thromboembolism and migraine with aura, were not measured by VIGITEL, 2008. For this reason, the prevalence of use of OCP along with contraindication may be underestimated. Data concerning other aspects of the reproductive history of women were also not available in this survey. These variables can potentially be determinants of the use of contraceptive methods, indicating that the final analysis can incorporate residual confusion.

The non-characterization of the type of OCP reported by the user in terms of their hormonal composition can be considered another potential limitation. OCP composed only of progestogens have contraindications only for more serious diseases. However, the use of this type of contraceptive is relatively low, probably because of the side effects, such as breakthrough bleeding, present in about 70% of women using this method^9^ ^,^ ^16^. Thus, the results of estimates of contraindicated use should be faced with caution and regarded as epidemiological marker events of their use.

The results of this study estimated a prevalence of almost 12% of use of OCP along with contraindications among Brazilian women in reproductive age. Education level was an inequity factor in the contraindicated use of OCP, and the group of women over 35 years presented itself as a potential surveillance group in their contraindicated use. This study may help identifying the possible existence of an undesirable iniquity in the reproductive health-care of Brazilian women.

The findings of this study point to the need for reviewing the activities and services concerning the reproductive planning in the Country, including counseling and free informed choice. Even with the availability of OCP in drugstores counters, one must empower women about their risks and benefits, as well as of other options for birth control. The identification of inequalities in the access and use of health services is focus of attention of academic researches and public administrators, for it provides important information to the planning of public health actions. Surveillance systems can help monitoring possible trends of the outcome studied.

## Footnotes

a Perpétuo MHO, Wong LLR. Desigualdade socioeconômica na utilização de métodos anticoncepcionais no Brasil: uma análise comparativa com base na PNDS 1996 a 2006. In: Ministério da Saúde. Pesquisa Nacional de Demografia e Saúde da Criança e da Mulher - PNDS 2006: dimensões do processo reprodutivo e da saúde da criança. Brasília (DF): Ministério da Saúde; 2009. Cap. 5.

b National Center for Chronic Disease Prevention and Health Promotion USA), Division of Reproductive Health. U.S. Selected practice recommendations for contraceptive use, 2013: adapted from de World Health Organization selected practice recommendations for contraceptive use, 2nd edition. MMWR Recomm Rep. 2013;62(RR05):1-46.

c World Health Organization. Medical eligibility criteria for contraceptive use. 4th ed. Geneva: WHO; 2009 [cited 2014 jul]. Available from: http://www.who.int/reproductivehealth/publications/family_planning/9789241563888/en/index.html

d Institute for Health Metrics and Evaluation (IHME). Global Burden of Disease (GBD) profile: Brazil. Seattle: IHME; 2013 [cited 2013 Apr 30). Available from: http://www.healthmetricsandevaluation.org/gbd/country-profiles

e Cabral CS. Socialização contraceptiva e o aprendizado da sexualidade: uma abordagem socioantropológica do processo de construção da prática contraceptiva. In: Anais do 18º Encontro Nacional de Estudos Populacionais; 13 a 23 de Novembro, 2012; Águas de Lindóia/SP-Brasil. Associação Brasileira de Estudos Populacionais; 2012.
